# Fine mapping of the male-sterile genes (*MS1*, *MS2*, *MS3*, and *MS4*) and development of SNP markers for marker-assisted selection in Japanese cedar (*Cryptomeria japonica* D. Don)

**DOI:** 10.1371/journal.pone.0206695

**Published:** 2018-11-15

**Authors:** Yoichi Hasegawa, Saneyoshi Ueno, Asako Matsumoto, Tokuko Ujino-Ihara, Kentaro Uchiyama, Satoko Totsuka, Junji Iwai, Tetsuji Hakamata, Yoshinari Moriguchi

**Affiliations:** 1 Graduate School of Science and Technology, Niigata University, Niigata, Niigata, Japan; 2 Department of Forest Molecular Genetics and Biotechnology, Forestry and Forest Products Research Institute, Forest Research and Management Organization, Tsukuba, Ibaraki, Japan; 3 Niigata Prefectural Forest Research Institute, Murakami, Niigata, Japan; 4 Forestry and Forest Products Research Center, Shizuoka Prefectural Research Institute of Agriculture and Forestry, Hamamatsu, Shizuoka, Japan; USDA/ARS, UNITED STATES

## Abstract

Pollinosis caused by Japanese cedar (*Cryptomeria japonica*) is a widespread social problem in Japan. To date, 23 male-sterile *C*. *japonica* trees have been selected in Japan to address pollinosis, from which four male-sterility loci (*MS1*, *MS2*, *MS3*, and *MS4*) have been identified from test crossing results. For efficient breeding of male-sterile *C*. *japonica* trees, more male-sterile individuals and individuals heterozygous for male-sterile genes are required. Therefore, we aimed to develop DNA markers for marker-assisted selection of four types of male-sterile genes from populations without a family structure. First, for four families exhibiting segregation of each male-sterile locus (*MS1*, *MS2*, *MS3*, and *MS4*), genome-wide single-nucleotide polymorphism and insertion/deletion (indel) genotyping was performed using the Axiom myDesign Targeted Genotyping Array method. Four high-density linkage maps for mapping the *MS1*, *MS2*, *MS3*, and *MS4* families were constructed, which included 4923, 1722, 1896, and 2247 markers, respectively. In these maps, 15, 4, 2, and 2 markers were located 0.0, 3.3, 1.1, and 0.0 cM from the *MS1*, *MS2*, *MS3*, and *MS4* loci, respectively. Second, for the markers located 0.0 cM from a male-sterile locus (i.e., *MS1* and *MS4*), to clarify the most tightly linked markers, we calculated the prediction rate of male-sterile gene genotypes from marker genotypes for 78 trees. The markers with the highest prediction rates were AX-174127446 (0.95) for *MS1* and AX-174121522 (1.00) for *MS4*. The AX-174121522 marker was considered to be suitable for selecting trees homozygous or heterozygous for the *MS4* gene from plus-trees without a pollination test, which requires a large amount of time and effort. The nearest markers to the male-sterile loci found in this study may facilitate the isolation of male-sterile genes in *C*. *japonica* via combination with the draft genomic sequence that is currently being collated.

## Introduction

Marker-assisted selection (MAS) is a breeding approach used to select individuals with desirable traits based on the genotypes of markers tightly linked to target genes, as has been reported for barley [1] and grape [2]. The main advantage of MAS is that it reduces the time required for breeding cycles. This is especially attractive for tree species with longer generation times than other crop species. MAS application is considered to be difficult in forest trees (especially conifers) due to the rapid decay of linkage disequilibrium [3]. However, in recent years, the construction of high-density linkage maps that facilitate the development of MAS markers has become more efficient and economical with the help of next-generation sequencing and high-throughput array technologies, even in non-model species.

Japanese cedar (*Cryptomeria japonica* D. Don) is an allogamous, monoecious, and wind-pollinated conifer with a haploid chromosome number (n) of 11 (2n = 22). The DNA content of the haploid cells was estimated to be 11.045 pg/C using flow cytometry, corresponding to a 10.8 Gb haploid genome size [4]. *C*. *japonica* is the most important forestry species in Japan, with outstanding features such as easy processes for cutting/propagation and artificial promotion of flowering, wide adaptability to the environment, rapid growth, straight bole, and soft wood with a pleasant color and scent. It covers an area of 4.5 million hectares, accounting for 44% of all Japanese artificial forests [5].

An increase in the area of *C*. *japonica* planted forests of has resulted in widespread *C*. *japonica* pollinosis, which is one of the most serious allergic diseases in Japan. In 2008, 26.5% of Japanese residents had an allergy to *C*. *japonica* pollen [6]. Genetically male-sterile *C*. *japonica* trees are expected to play an important role in reducing the amount of pollen dispersed by the breeding program. The first genetically male-sterile *C*. *japonica* tree, Toyama-1, the sterility of which is controlled by the *MS1* locus [7,8], was discovered in 1992 [9]. There has since been considerable effort to characterize male sterility in *C*. *japonica*, for example, by identifying male-sterile individuals in plus-tree populations, making artificial matings between male-sterile and plus-trees, and propagating male-sterile trees. To date, 23 genetically male-sterile trees have been identified from seven prefectures [8], and the frequency of male-sterile trees in artificial forests has been estimated as one in several thousand [10,11]. Based on the results of test crossings, four recessive male-sterile genes, *MS1*, *MS2*, *MS3*, and *MS4*, were identified [7,8,12,13].

Male-sterile trees are currently identified by observing the release of pollen or by directly examining male strobili with a magnifying glass or microscope. Heterozygotes of male-sterile genes have been identified by examining the segregation data of the progeny arising from artificial crosses. Such trees are important breeding material for the production of male-sterile *C*. *japonica* trees. Most plus-trees heterozygous for a male-sterile gene found in Japan (e.g., Suzu-2, Ooi-7, Naka-4, and Ohara-13) are controlled by *MS1* because test crossing has been performed using Toyama-1. Therefore, heterozygous plus-trees for a male-sterile gene controlled by *MS2*, *MS3*, and *MS4* are required as breeding materials to avoid problems associated with inbreeding depression. Because conventional methods require time, labor, and space, it is desirable to localize the male-sterile genes on the linkage map and develop tightly linked DNA markers for MAS. Numerous DNA markers have been developed to promote the molecular breeding of *C*. *japonica* [14–23]. In addition, linkage maps were constructed with 1262 markers (average marker interval: 1.1 cM [24]), 2560 markers (0.49 cM [25]), and 3205 markers (0.47 cM [23]). Using information from these linkage maps, the male-sterile genes *MS1*, *MS2*, *MS3*, and *MS4* have been mapped onto the ninth linkage group (hereafter denoted as LG9 [24]), the fifth linkage group (LG5 [19]), the first linkage group (LG1 [25]), and the fourth linkage group (LG4 [25]), respectively.

In most high-density maps, male-sterile loci are not mapped due to the use of families unrelated to male sterility. In this study, we developed a large number of new single-nucleotide polymorphism (SNP) and insertion/deletion (indel) markers and constructed four high-density linkage maps exhibiting consistent marker order including the *MS1*, *MS2*, *MS3*, and *MS4* genes. Furthermore, we calculated the prediction rate of male-sterile gene genotypes from marker genotypes in a population without a family structure. These results allowed us to develop markers for MAS of male-sterile genes from plus-trees with superior traits (i.e., outside the mapping population).

## Materials and methods

### Ethics statement

All needle tissues used in this study were collected from scion gardens in Forestry and Forest Products Research Institute, Niigata University and five Prefectural Forest Experiment Stations (Niigata, Shizuoka, Ishikawa, Kanagawa, and Fukushima). Permission is obtained from all Institutes. In addition, plant tissues of natural forests were collected from the scion garden established using cutting with permission.

### Reference transcript construction

Reference transcripts were constructed by clustering three sets of transcriptomes. Two sets were from RNA sequencing experiments (Ueno et al. [26] and this study) and one from full-length cDNA libraries [27]. Three sets of transcript sequences (totaling 573,944 sequences) were clustered with EvidentialGene [28], resulting in 81,510 “okay” and “okalt” transcripts (EviGene transcripts). We added 16 male sterility candidate gene homologues in *C*. *japonica* (Futamura, personal communication), because they were not completely represented in the EviGene transcripts (HSP for candidate gene homolog/HSP for EviGene transcript ≤ 0.6, where HSP indicates the high-scoring segment pair in a BLAST search). In addition, 49 candidate genes detected in a genome-wide association study by Uchiyama et al. [29] were added, because they were not completely represented in the EviGene transcripts (HSP for candidate gene homolog/HSP for EviGene transcript ≤ 0.6). We used these 81,575 sequences with a mean length of 1128 bp as a reference for SNP and indel discovery.

### RNA extraction, sequencing, and SNP and indel discovery

Four male-fertile trees with heterozygous male-sterile genes (Ooi-7 [*Ms1/ms1*], S1NK4 [*Ms2/ms2*], S5HK7 [*Ms3/ms3*], and S8HK5 [*Ms4/ms4*]) were used to develop SNP and indel markers using RNA sequencing. Ooi-7 was a plus-tree heterozygous for a male-sterile gene (*MS1*) found in Shizuoka Prefecture [30]. S1NK4 was an F_1_ of Shindai-1 (male-sterile, *ms2/ms2*) and Nakakubiki-4 (male-fertile, *Ms2/Ms2*), where male sterility in Shindai-1 was controlled by the *MS2* locus [12]. S5HK7 was an F_1_ of Shindai-5 (male-sterile, *ms3/ms3*) and Higashikanbara-7 (male-fertile, *Ms3/Ms3*), where male sterility in Shindai-5 was controlled by the *MS3* locus [12]. S8HK5 was an F_1_ of Shindai-8 (male-sterile, *ms4/ms4*) and Higashikanbara-5 (male-fertile, *Ms4/Ms4*), where male sterility of Shindai-8 was controlled by the *MS4* locus [13]. For S1NK4 and S5HK7, RNA was extracted from the male strobili, inner bark, and needles. However, for Ooi-7 and S8HK5, RNA was extracted from only the inner bark and needles. Total RNA was extracted based on the method of Le Provost et al. [31]. Approximately ten spoonfuls of powder were added to 1 mL of CTAB extraction buffer. Extracted RNA was further purified with an SV total RNA purification system (Promega, Madison, WI, USA). Sequencing libraries were constructed and sequenced using a HiSeq 2500 (Illumina, San Diego, CA, USA) in 2 × 100-bp paired-end sequencing by Hokkaido System Science Co., Ltd (Sapporo, Japan). SNP and indel discovery was performed using Genedata Expressionist Genomic Profiling 9.1.4a software (Genedata, Lexington, MA, USA) at Takara Bio Inc. (Otsu, Shiga, Japan). First, 441 million reads were obtained by removing low-quality regions and sequence adaptor-derived regions using Trimmomatic 0.32 [32]. Following removal, sequence reads were mapped to the reference sequences described in the previous section using BWA-MEM 0.7.12 [33]. Low-quality and duplicate reads were removed from the mapping results and realigned. The differences between the reference sequence and read sequence were detected as genetic mutations. Of the 996,871 SNP and 91,825 indel mutations, 775,550 SNP and 64,807 indel mutations were heterozygous. The 69,199 heterozygous SNPs and 4235 heterozygous indels were used for Axiom myDesign Targeted Genotyping Array (Affymetrix; Thermo Fisher Scientific, Waltham, MA, USA).

### DNA extraction and axiom genotyping

High-density linkage maps of *C*. *japonica* were constructed for the F1O7 (84 individuals), S1-2 (94 individuals [19,25]), S5HK7 (94 individuals [25]), and S8HK5 (96 individuals [25]) families (Table 1). The F1O7 family was derived from a cross between Fukushima-1 (male-sterile, *ms1/ms1*) and Ooi-7, where male sterility in Fukushima-1 is controlled by the *MS1* locus [8,10]. The S1-2 family was derived from a backcross between Shindai-1 and the F_1_ plant S1NK4. The S5HK7 family was derived from a backcross between Shindai-5 and the F_1_ plant S5HK7. Finally, the S8HK5 family was derived from a backcross between Shindai-8 and the F_1_ plant S8HK5.

**Table 1:** Summary of the families used to construct the linkage maps in this study.

Family	Number of	Maternal plant	Paternal plant
name	individuals		
F1O7	84	Fukushima-1 [*ms1*/*ms1*]	Ooi-7 [*Ms1*/*ms1*]
S1-2	94	Shindai-1 [*ms2*/*ms2*]	S1NK4 (Shindai-1 × Nakakubiki-4) F_1_ [*Ms2*/*ms2*]
S5HK7	94	Shindai-5 [*ms3*/*ms3*]	S5HK7 (Shindai-5 × Higashikanbara-7) F_1_ [*Ms3*/*ms3*]
S8HK5	96	Shindai-8 [*ms4*/*ms4*]	S8HK5 (Shindai-8 × Higashikanbara-5) F_1_ [*Ms4*/*ms4*]

In addition to the progeny and parents of the four mapping populations, four male-sterile trees, seven trees heterozygous for male-sterile genes, parents of the previous linkage map, seven trees from breeding materials such as plus-trees and cutting cultivars, and 50 trees from natural forests were used to calculate the prediction rate of male-sterile gene genotypes from marker genotypes (for a total of 78 trees; S1 Table).

Needle tissue was collected from all 446 trees (i.e., four mapping populations and 78 trees previously mentioned). Genomic DNA was extracted from these needles using a modified CTAB method [34]. Using Axiom myDesign Targeted Genotyping Array with 73,434 markers (GPL25617), information on the genotypes of the 446 trees was obtained according to the manufacturer’s instructions. Genotyping was performed using Axiom Analysis Suite ver. 2.0.0.35 software. Markers with an off-target variant or Fisher’s linear discriminant < 7 were not used for subsequent analysis.

### Linkage map construction

Chi-squared tests were performed for each locus to assess the deviation from the expected Mendelian segregation ratio. Loci showing extreme segregation distortion (*P* < 0.001) and with many missing data points (more than three individuals) were excluded from further linkage analysis.

The linkage analyses were performed using the maximum likelihood mapping algorithm of JoinMap ver. 4.1 software (Kyazma, Wageningen, The Netherlands) with a cross pollination (CP)-type population (i.e., *hk* × *hk*, *lm* × *ll*, and *nn* × *np*) for the F1O7 family and backcross (BC)-type population (i.e., *nn* × *np*) for the S1-2, S5HK7, and S8HK5 families. The resulting 4923, 1722, 1897, and 2247 markers were used for linkage analysis of the F1O7, S1-2, S5HK7, and S8HK5 families, respectively. In all mapping populations, the trait data of male-sterile and male-fertile trees were recorded as homozygous and heterozygous genotypes, respectively, according to the method of Moriguchi et al. [19,24,25] (S2–S5 Tables). Markers were initially assigned to tentative linkage groups using logarithm of odds ratio (LOD) thresholds of 3.0–9.0 in increments of 1.0. LOD thresholds of 6.0, 4.0, 4.0, and 5.0 for groups of markers were defined for the F1O7, S1-2, S5HK7, and S8HK5 families, respectively. Map distance was calculated using the Kosambi mapping function. Default settings were used for the recombination frequency threshold and ripple value. The linkage group numbers (LG1–LG11) were defined following the linkage maps published by Tani et al. [35] and Moriguchi et al. [24]. Images of the linkage groups were drawn using MapChart ver. 2.0 [36].

### Prediction rate of male-sterile gene genotypes from marker genotypes

We found 15 and 2 markers located 0.0 cM from the *MS1* and *MS4* genes, respectively (see Results and discussion). To clarify the most tightly linked markers, we calculated the prediction rate of male-sterile gene genotypes from marker genotypes for 78 trees. The prediction rate was defined as the ratio of the number of individuals with no inconsistencies between marker genotype and the genotype of the male-sterility locus to the number of all individuals.

## Results and discussion

### High-density linkage maps

In total, 10,808 (F1O7 family), 11,442 (S1-2 family), 10,882 (S5HK7 family), and 10,913 (S8HK5 family) of the 73,434 markers on the Axiom array were successfully genotyped in this study (Table 2). In the F1O7 family (CP type), 4959 markers showed polymorphisms. In S1-2, S5HK7, and S8HK5 families (BC types), 1742, 2118, and 2363 markers showed polymorphisms, respectively. After excluding markers with large amounts of missing data and high distortions in the segregation ratio, 4923 (F1O7 family), 1719 (S1-2 family), 1897 (S5HK7 family), and 2247 (S8HK5 family) markers were ultimately used for the linkage analysis. The total length of the linkage maps constructed in this study for the F1O7, S1-2, S5HK7, and S8HK5 families was 1575.5, 1290.1, 1168.6, and 2350.9 cM and the average interval between markers was 0.32, 0.75, 0.62, and 1.05 cM, respectively (S1–S4 Figs and S2–S5 Tables). These maps had the shortest mean distance between adjacent markers in the *C*. *japonica* linkage map, including the male sterility locus, reported to date. In addition, these values were similar to the linkage maps of other conifers (e.g., 0.58 cM/marker in *Pinus taeda* [37], 0.60 cM/marker in a consensus map for *P*. *taeda* and *Pinus elliottii* [38], 0.93 cM/marker in *Pinus pinaster* [39], and 0.92 cM/marker in a consensus map for *Picea glauca* and *Pinus marina* [40]). Since the high-density linkage maps were constructed using markers derived from the cDNA libraries of *C*. *japonica*, they are useful not only for quantitative trait locus (QTL) analysis of this species, but also for chromosomal synteny analysis across related species of Cupressaceae.

**Table 2:** Numbers of markers used to construct the four linkage maps.

Family	Number of	Number of markers	Number of polymorphic markers between parents		Number of markers	Number of
name	analyzed markers	successfully genotyped	CP type	BC type	used for linkage analyses	positioned markers
F1O7	73,434	10,808	4,959	-	4,923	4,923
S1-2	73,434	11,442	-	1,742	1,719	1,719
S5HK7	73,434	10,882	-	2,118	1,897	1,896
S8HK5	73,434	10,913	-	2,363	2,247	2,247

**Fig 1:**
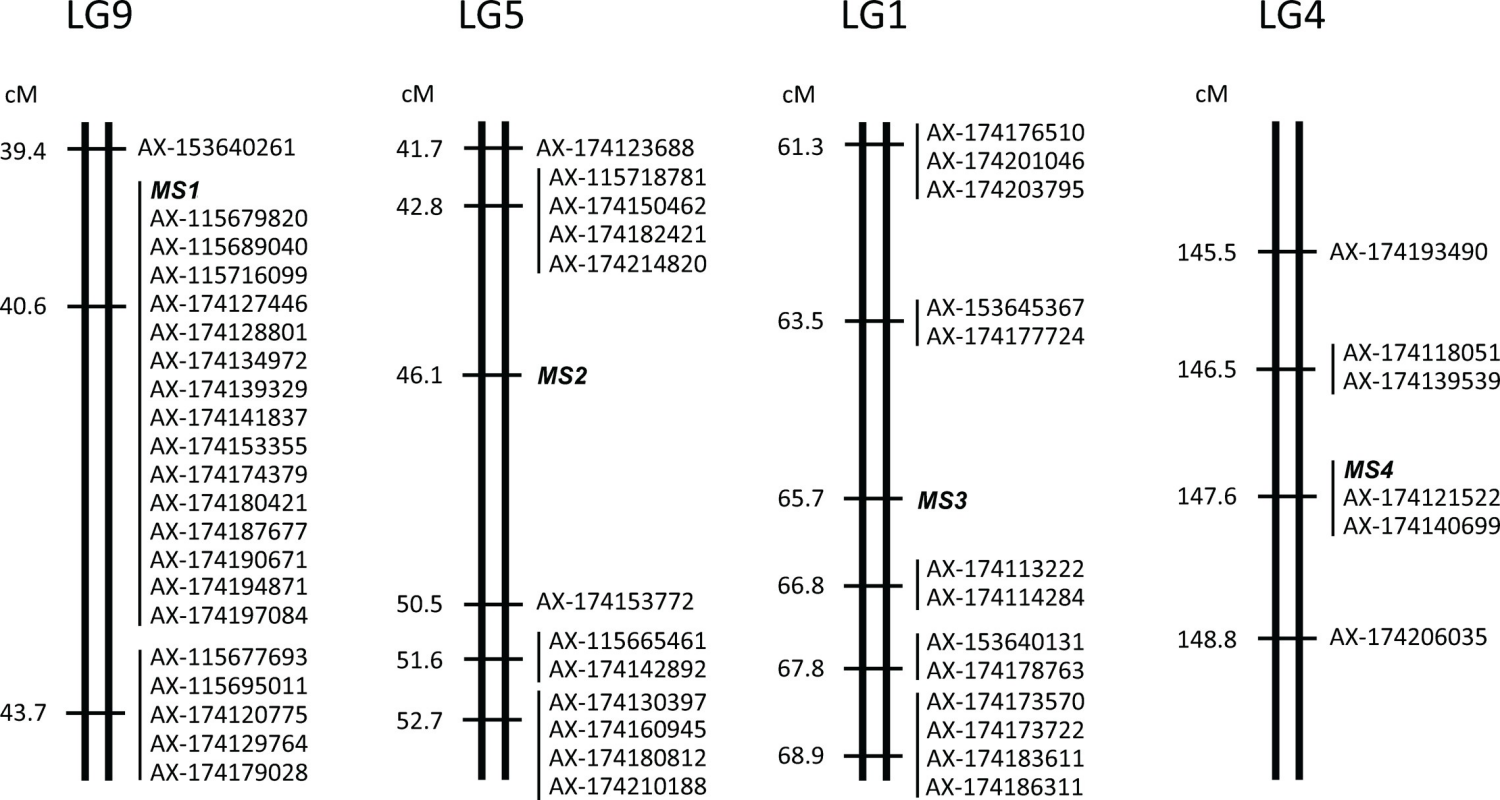
Partial linkage maps around the male-sterile genes *MS1*, *MS2*, *MS3*, and *MS4*. Marker names are indicated to the right of the linkage groups. Centimorgan distances are indicated to the left of each linkage group. Full linkage maps are shown in S1–S4 Figs.

In total, 15, 4, 2, and 2 markers were mapped to the vicinities of the *MS1*, *MS2*, *MS3*, and *MS4* loci, respectively (Fig 1). These markers were separated from their associated male-sterility genes by 0.0, 3.3, 1.1, and 0.0 cM, respectively, in the F1O7, S1-2, S5HK7, and S8HK5 families. Several other studies have attempted to develop markers closely linked to male-sterile genes in *C*. *japonica*. Moriguchi et al. [25] reported the closely linked markers for four male-sterile genes, in which the closest markers to the *MS1*, *MS2*, *MS3*, and *MS4* male-sterile loci were estSNP04188 (1.8 cM in the T5 family of 173 individuals), estSNP00695 (7.0 cM in the S1-2 family of 128 individuals), gSNP05415 (3.1 cM in the S5HK7 family of 167 individuals), and estSNP01408 (7.0 cM in the S8HK5 family of 108 individuals), respectively. In addition, Mishima et al. [23] reported markers that overlapped in the QTL region for *MS1*. Although some markers reported in this study were closer than those reported previously (see below), they are difficult to compare directly since map distance depends on the number of individuals of a mapping family. Therefore, all of these closely linked markers should be mapped using the same families with a large number of individuals to investigate their accurate map positions in future studies. Determining the precise map distance of closely linked markers could enable more precise MAS selection by using two markers sandwiching a male-sterile locus.

### Prediction rate of male-sterile gene genotypes from marker genotypes

We identified markers located 0.0 cM from the *MS1* and *MS4* genes and calculated the prediction rates of the male-sterile gene genotype based on the genotypes of these markers using 78 individual trees without a family structure (S1 Table). The prediction rates for the 15 markers 0 cM from the *MS1* gene were 0.29–0.95 (Table 3). Meanwhile, the prediction rates of the two markers 0 cM from the *MS4* gene were 0.69 and 1.00. The highest prediction rates for the *MS1* and *MS4* genes were 0.95 (AX-174127446) and 1.00 (AX-174121522), respectively (S5 and S6 Figs), and the prediction rates of these markers were the highest among all 73,434 markers in this study (S6 Table).

**Table 3:** Positions of the markers closest to the male-sterile genes and prediction rate of male-sterile gene genotypes from the marker genotypes of 78 *Cryptomeria japonica* trees.

Family name	Locus name		Linkage maps	Populations without family structure
(Target male-sterile gene)		Position	Map distance from	Prediction rate of male-sterile gene
		(cM)	*MS* gene (cM)	genotypes from the marker genotypes
F1O7 (*MS1*)	AX-115679820	40.6	0.0	0.32
	AX-115689040	40.6	0.0	0.82
	AX-115716099	40.6	0.0	0.65
	AX-174127446	40.6	0.0	0.95
	AX-174128801	40.6	0.0	0.53
	AX-174134972	40.6	0.0	0.35
	AX-174139329	40.6	0.0	0.69
	AX-174141837	40.6	0.0	0.82
	AX-174153355	40.6	0.0	0.78
	AX-174174379	40.6	0.0	0.33
	AX-174180421	40.6	0.0	0.32
	AX-174187677	40.6	0.0	0.58
	AX-174190671	40.6	0.0	0.41
	AX-174194871	40.6	0.0	0.47
	AX-174197084	40.6	0.0	0.29
S1-2 (*MS2*)	AX-115718781	43.9	3.3	-
	AX-174150462	43.9	3.3	-
	AX-174182421	43.9	3.3	-
	AX-174214820	43.9	3.3	-
S5HK7 (*MS3*)	AX-174113222	66.8	1.1	-
	AX-174114284	66.8	1.1	-
S8HK5 (*MS4*)	AX-174121522	147.6	0.0	1.00
	AX-174140699	147.6	0.0	0.69

The 78 trees included eight parents of the four mapping populations, four male-sterile trees, seven trees heterozygous for a male-sterile gene, two parents of the previous linkage map, seven trees from breeding materials, and 50 trees from natural forests (see S1 Table).

We did not calculate the prediction rates for the *MS2* and *MS3* genes, because the markers are located 0.0 cM from the *MS2* and *MS3* genes. To develop markers that are more tightly linked to the *MS2* and *MS3* genes used for MAS, it is necessary to map more markers in the S1-2 and S5HK7 families.

The AX-174121522 marker was considered to be suitable for selection of trees homozygous or heterozygous for the *MS4* gene from plus-trees without test crossing, which requires a large amount of time and effort. The BLAST search revealed that cDNA containing a SNP of AX-174121522 was similar to the uroprophyrinogen-III synthase gene. With respect to the nucleotide substitution of the SNP, the amino acid substitution from threonine to asparagine was confirmed. Therefore, it is possible that the male-sterility of *MS4* is caused by nucleotide substitution of the SNP (AX-174121522), and further experiments are necessary to clarify whether this SNP mutation is responsible for male-sterility in *MS4*. For AX-17412746 (the closest marker to *MS1* in this study), the rate of prediction was not 1.00. Mishima et al. [23] reported two markers closely linked to the *MS1* locus (reCj19250_1927 and reCj19250_2335) in the sosyun family. The genotypes of these markers (G/G in reCj19250_1927 and C/C in reCj19250_2335) were not consistent with the expected genotype from the phenotype (G/A in reCj19250_1927 and C/T in reCj19250_2335) in the Ooi-7 used in this study. Therefore, these three markers appeared to be separated by some distance from the *MS1* gene. We are currently generating the draft genome sequence of *C*. *japonica*, and cumulative information for the high-quality draft genome sequence and closely linked markers to male-sterile genes might facilitate the isolation of male-sterile genes in *C*. *japonica*.

## Supporting information

S1 Fig Linkage map for *Cryptomeria japonica* derived from the F1O7 family (LG1–LG11). Marker names are indicated to the right of the linkage groups. Centimorgan distances are indicated to the left of each linkage group.
(PDF)

S2 Fig Linkage map for *Cryptomeria japonica* derived from the S1-2 family (LG1–LG11). Marker names are indicated to the right of the linkage groups. Centimorgan distances are indicated to the left of each linkage group.
(PDF)

S3 Fig Linkage map for *Cryptomeria japonica* derived from the S5HK7 family (LG1–LG11). Marker names are indicated to the right of the linkage groups. Centimorgan distances are indicated to the left of each linkage group.
(PDF)

S4 Fig Linkage map for *Cryptomeria japonica* derived from the S8HK5 family (LG1–LG11). Marker names are indicated to the right of the linkage groups. Centimorgan distances are indicated to the left of each linkage group.
(PDF)

S5 Fig Genotypes of the male-sterile gene (*MS1*) and markers obtained using Affymetrix microarray. Genotypes with red backgrounds showed inconsistencies between the marker genotypes and genotype for the *MS1* locus.
(PDF)

S6 Fig Genotypes of the male-sterile gene (*MS4*) and markers obtained using Affymetrix microarray. Genotypes with red backgrounds showed inconsistencies between the marker genotypes and genotype for the *MS4* locus.
(PDF)

S1 Table Summary of the *Cryptomeria japonica* trees used to calculate the prediction rate of male-sterile gene genotypes from the marker genotypes. (XLSX)

S2 Table Positions of mapped markers and the *MS1* gene and these genotypes of the F1O7 family. Individual name with ms indicates the male sterility.
(XLSX)

S3 Table Positions of mapped markers and the *MS2* gene and these genotypes of the S1-2 family. Individual name with ms indicates the male sterility.
(XLSX)

S4 Table Positions of mapped markers and the *MS3* gene and these genotypes of the S5HK7 family. Individual name with ms indicates the male sterility.
(XLSX)

S5 Table Positions of mapped markers and the *MS4* gene and these genotypes of the S8HK5 family. Individual name with ms indicates the male sterility.
(XLSX)

S6 Table Prediction rate of male-sterile gene genotypes from the marker genotypes of 78 *Cryptomeria japonica* trees. The 78 trees included eight parents of the four mapping populations, four male-sterile trees, seven trees heterozygous for male-sterile gene, two parents of the previous linkage maps, seven trees from breeding materials, and 50 trees from natural forests (see S1 Table).
(XLSX)
