# Supplementary material for: Fine mapping of the male-sterile genes (MS1, MS2, MS3, and MS4) and development of SNP markers for marker-assisted selection in Japanese cedar (Cryptomeria japonica D. Don)
Source: PLoS One. 2018 Nov 15;13(11):e0206695. doi: 10.1371/journal.pone.0206695 (PMC6237302; doi:10.1371/journal.pone.0206695)
Supplement: S1 Fig — Marker names are indicated to the right of the linkage groups. Centimorgan distances are indicated to the left of each linkage group. (PDF) [file pone.0206695.s001.pdf]

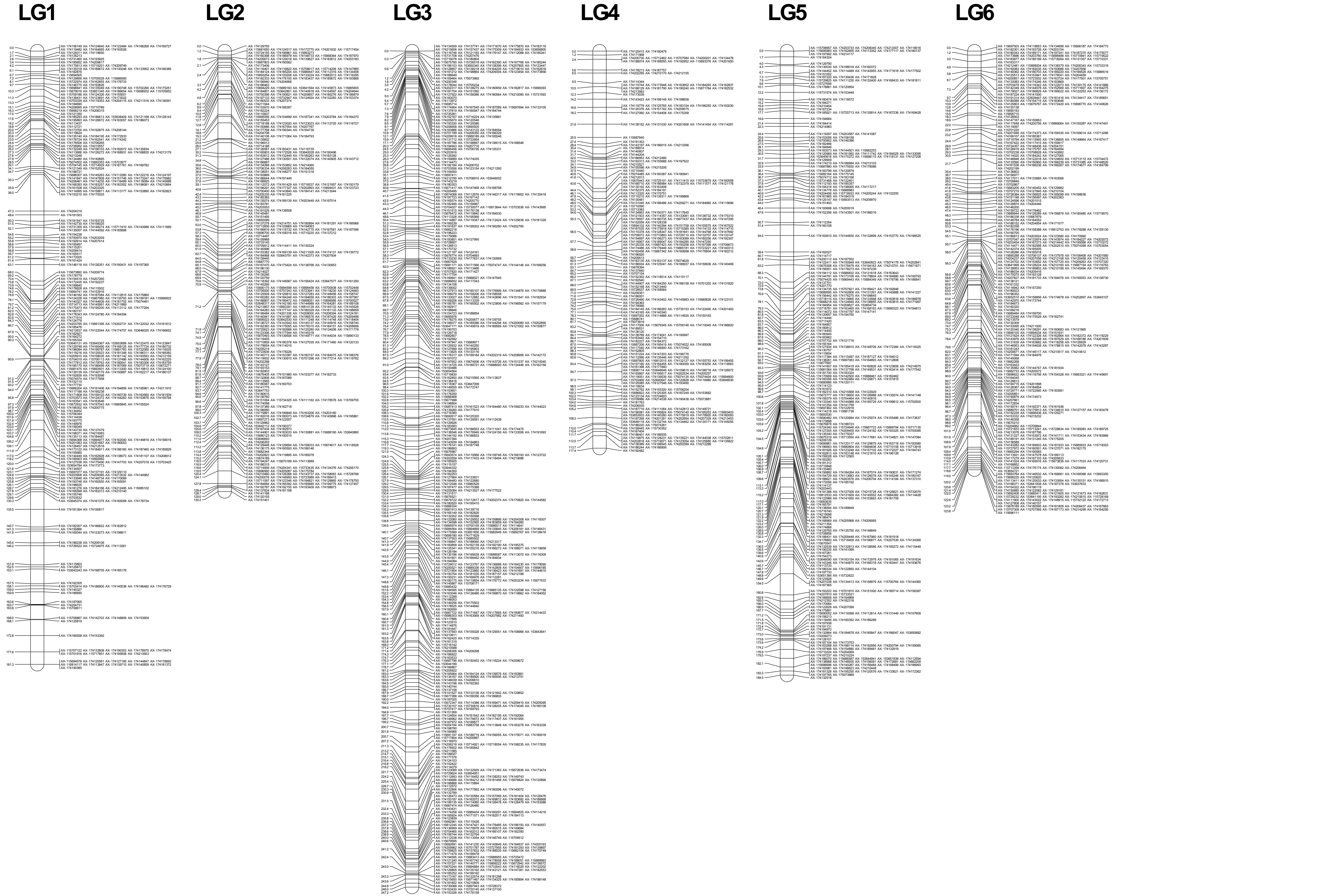

S1 Fig. Linkage map for *Cryptomeria japonica* derived from the F1O7 family (LG1–LG11). Marker names are indicated to the right of the linkage groups. Centimorgan distances are indicated to the left of each linkage group.

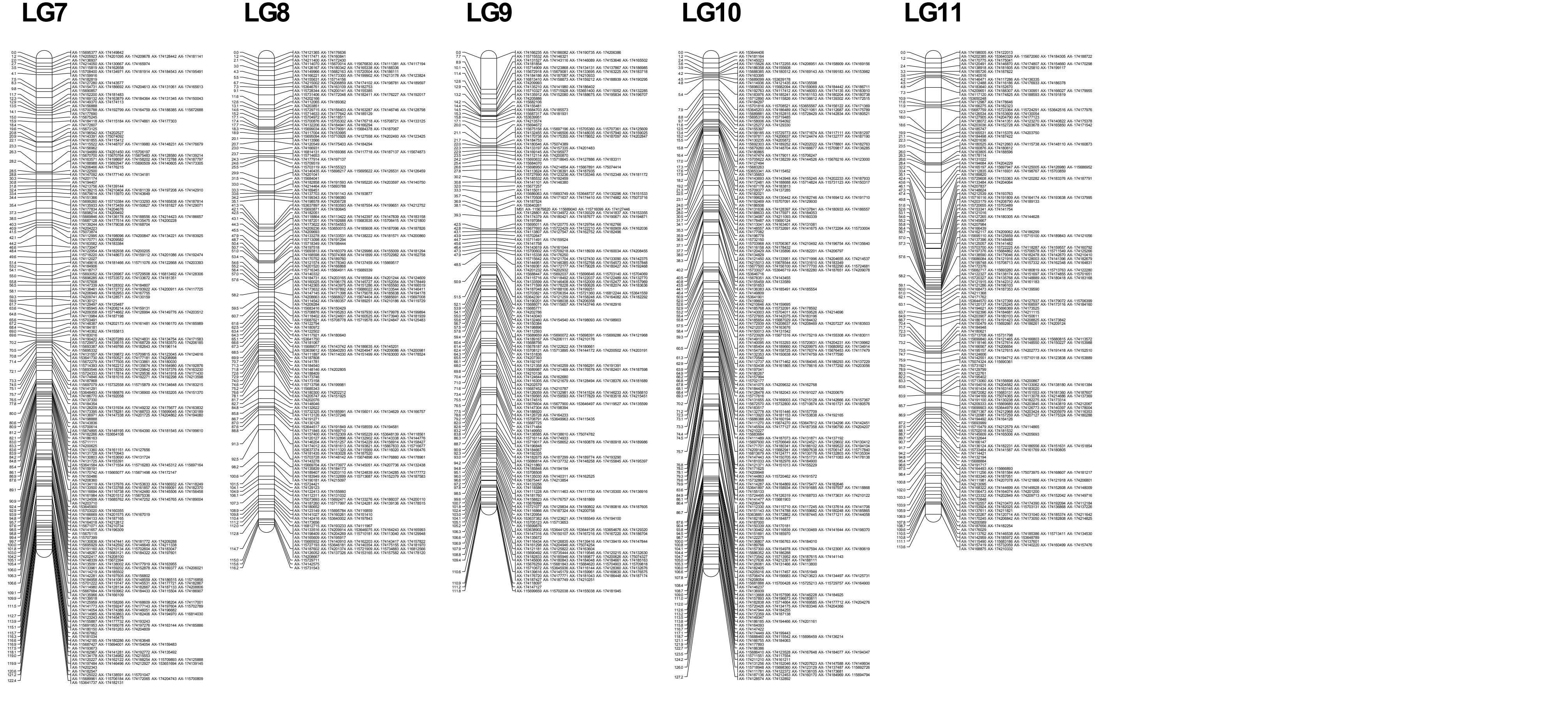

S1 Fig. (continued)
