# Supplementary material for: Fine mapping of the male-sterile genes (MS1, MS2, MS3, and MS4) and development of SNP markers for marker-assisted selection in Japanese cedar (Cryptomeria japonica D. Don)
Source: PLoS One. 2018 Nov 15;13(11):e0206695. doi: 10.1371/journal.pone.0206695 (PMC6237302; doi:10.1371/journal.pone.0206695)
Supplement: S2 Fig — Marker names are indicated to the right of the linkage groups. Centimorgan distances are indicated to the left of each linkage group. (PDF) [file pone.0206695.s002.pdf]

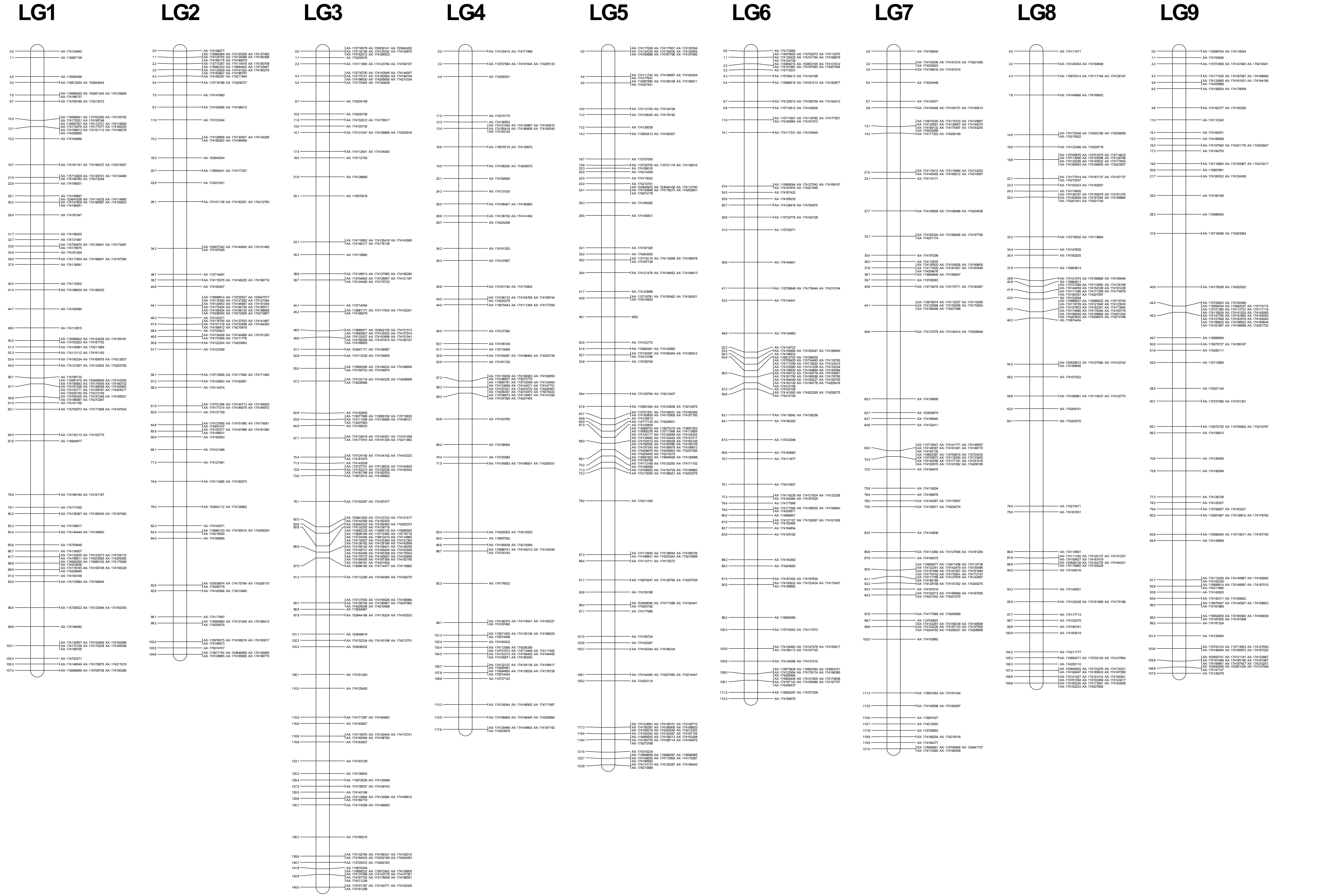

S2 Fig. Linkage map for *Cryptomeria japonica* derived from the S1-2 family (LG1–LG11). Marker names are indicated to the right of the linkage groups. Centimorgan distances are indicated to the left of each linkage group.

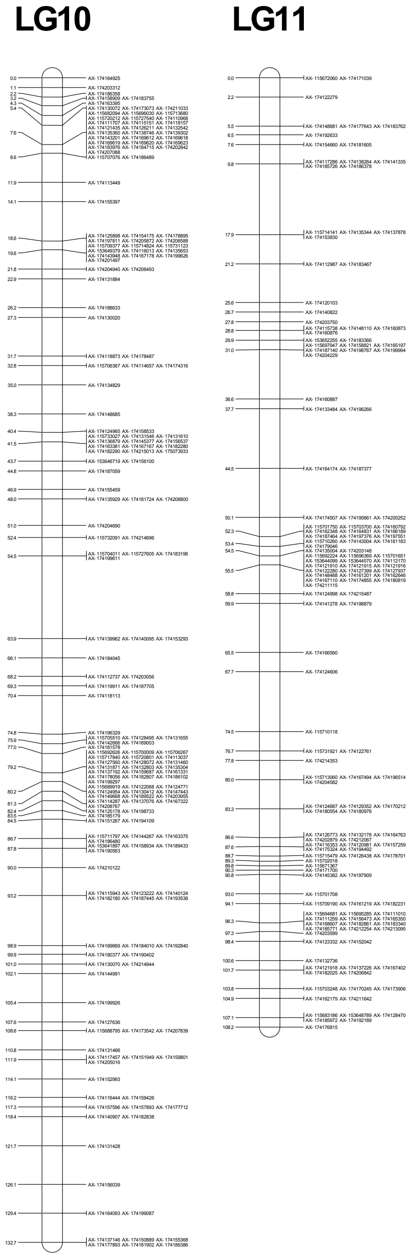

S2 Fig. (continued)
