# Supplementary figures and images for: Fine mapping of the male-sterile genes (MS1, MS2, MS3, and MS4) and development of SNP markers for marker-assisted selection in Japanese cedar (Cryptomeria japonica D. Don)
Source: PLoS One. 2018 Nov 15;13(11):e0206695. doi: 10.1371/journal.pone.0206695 (PMC6237302; doi:10.1371/journal.pone.0206695)

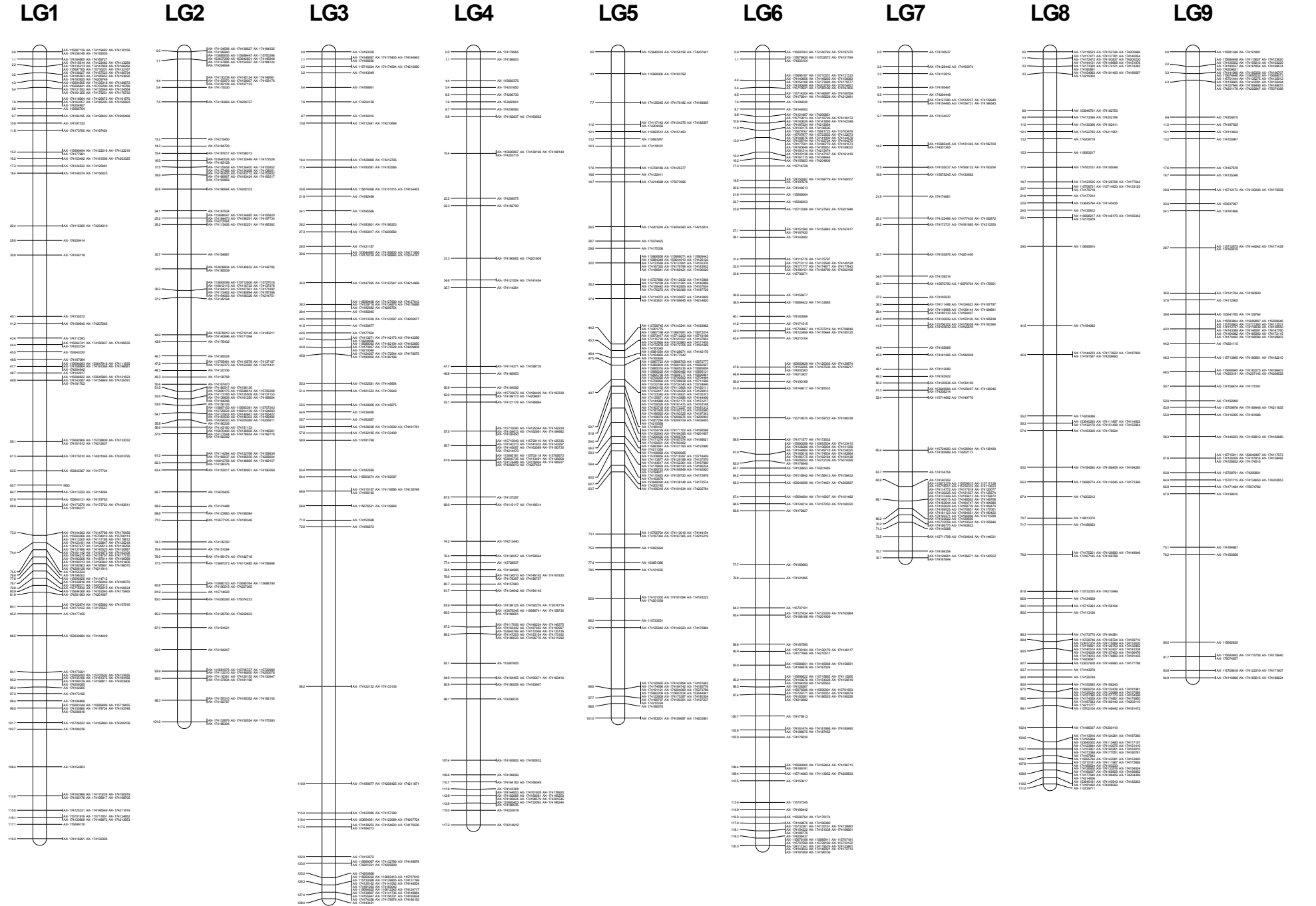

## LG10

## LG11

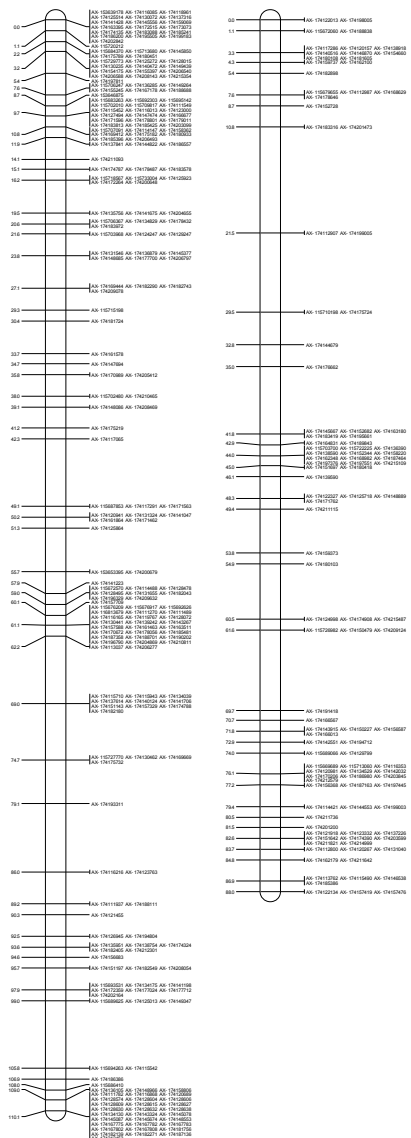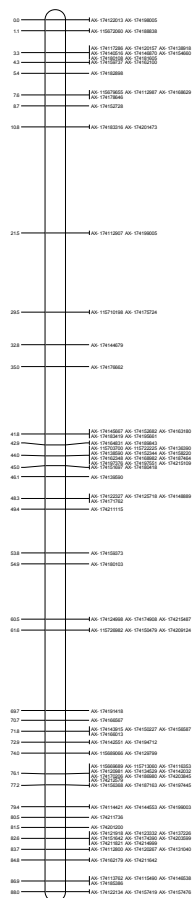

Supplement: S3 Fig — Marker names are indicated to the right of the linkage groups. Centimorgan distances are indicated to the left of each linkage group. (PDF) [file pone.0206695.s003.pdf]

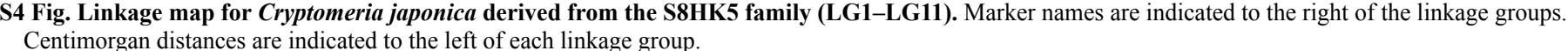

LG10

LG11

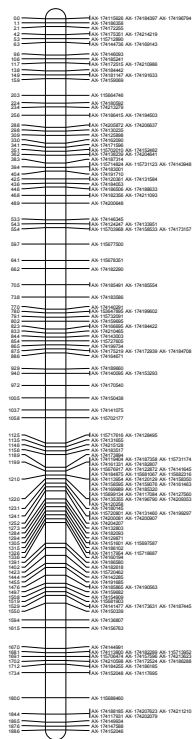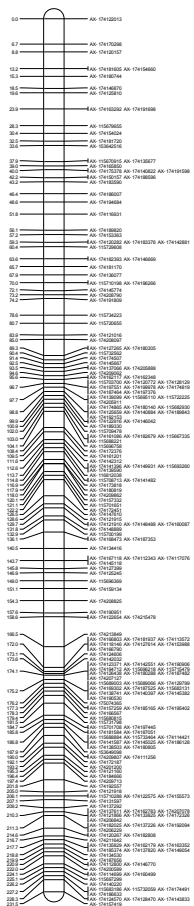

Supplement: S4 Fig — Marker names are indicated to the right of the linkage groups. Centimorgan distances are indicated to the left of each linkage group. (PDF) [file pone.0206695.s004.pdf]
