# Supplementary material for: Fine mapping of the male-sterile genes (MS1, MS2, MS3, and MS4) and development of SNP markers for marker-assisted selection in Japanese cedar (Cryptomeria japonica D. Don)
Source: PLoS One. 2018 Nov 15;13(11):e0206695. doi: 10.1371/journal.pone.0206695 (PMC6237302; doi:10.1371/journal.pone.0206695)
Supplement: S6 Fig — Genotypes with red backgrounds showed inconsistencies between the marker genotypes and genotype for the MS4 locus. (PDF) [file pone.0206695.s006.pdf]

Locus name	Shindai-3	Shindai-E	Shindai-T	Fukushima-1	Fukushima-2	Ooi-7	Naka-4	Suzu-2	Shindai-1	Shindai-1 x Nakakubiki-4	Niigata-2	Shindai-5	Shindai-5 x Higashikanbara-7	Niigata-31	Niigata-32	Niigata-33	Shindai-8	Shindai-8 x Higashikanbara-5	Niigata-4	YI-38
MS4	AA	AA	AA	AA	AA	AA	AA	AA	AA	AA	AA	AA	AA	AA	AA	AA	BB	AB	AB	AA
AX-174121522	AA	AA	AA	AA	AA	AA	AA	AA	AA	AA	AA	AA	AA	AA	AA	AA	BB	AB	AB	AA
AX-174140699	AB	AA	AA	AA	AA	AB	AB	AB	AA	AA	AA	AA	AA	AA	AA	AB	BB	AB	AB	AA

Locus name	YI-96	Higashikanbara-5	Iwao	Kunisaki-3	Nakakubiki-4	Shinobu-1	Yabukuguri	Kumotooshi	Ajigasawa-02	Ajigasawa-12	Ajigasawa-16	Ajigasawa-17	Ajigasawa-20	Ajigasawa-25	Ajigasawa-31	Ajigasawa-33	Ajigasawa-34	Ashu-01	Ashu-03	Ashu-13
MS4	AA	AA	AA	AA	AA	AA	AA	AA	AA	AA	AA	AA	AA	AA	AA	AA	AA	AA	AA	AA
AX-174121522	AA	AA	AA	AA	AA	AA	AA	AA	AA	AA	AA	AA	AA	AA	AA	AA	AA	AA	AA	AA
AX-174140699	AB	AA	AB	AA	AA	AA	AA	AA	AA	AA	AA	AA	AB	AA	AA	AA	AA	AA	AA	AA

Locus name	Ashu-14	Ashu-17	Ashu-21	Ashu-24	Ashu-25	Azouji-06	Azouji-08	Azouji-19	Azouji-22	Azouji-24	Azouji-25	Azouji-34	Azouji-40	Ishinomaki-01	Ishinomaki-04	Ishinomaki-07	Ishinomaki-10	Ishinomaki-17	Ishinomaki-18	Kawazu-01
MS4	AA	AA	AA	AA	AA	AA	AA	AA	AA	AA	AA	AA	AA	AA	AA	AA	AA	AA	AA	AA
AX-174121522	AA	AA	AA	AA	AA	AA	AA	AA	AA	AA	AA	AA	AA	AA	AA	AA	AA	AA	AA	AA
AX-174140699	AA	BB	AA	AB	AA	AB	AA	AA	AA	AA	AB	AA	AA	AB	AA	AA	AB	AA	AB	AA

Locus name	Kawazu-02	Kawazu-03	Kawazu-06	Kawazu-08	Kawazu-09	Land-07	Land-19	Land-25	Land-32	Shimowada-16	Shimowada-24	Shimowada-29	Shimowada-31	Shimowada-32	Yakushima-02	Yakushima-04	Yakushima-06	Yakushima-07
MS4	AA	AA	AA	AA	AA	AA	AA	AA	AA	AA	AA	AA	AA	AA	AA	AA	AA	AA
AX-174121522	AA	AA	AA	AA	AA	AA	AA	AA	AA	AA	AA	AA	AA	AA	AA	AA	AA	AA
AX-174140699	AA	AA	AB	AA	AA	AB	AB	BB	AB	AB	AA	AA	AB	AA	AA	AA	AB	AB

S6 Fig. Genotypes of the male-sterile gene (*MS4*) and markers obtained using Affymetrix microarray. Genotypes with red backgrounds showed inconsistencies between the marker genotypes and genotype for the *MS4* locus.
